# Synthesis of water-degradable silica nanoparticles from carbamate-containing bridged silsesquioxane precursor†

**DOI:** 10.1039/c7ra12377a

**Published:** 2018-01-29

**Authors:** Zhe Gao, Seyyed Pouya Hadipour Moghaddam, Hamidreza Ghandehari, Ilya Zharov

**Affiliations:** Department of Chemistry, University of Utah Salt Lake City UT 84112 USA i.zharov@utah.edu; Department of Pharmaceutics and Pharmaceutical Chemistry, College of Pharmacy, University of Utah USA; Utah Center for Nanomedicine, Nano Institute of Utah, University of Utah USA; Department of Bioengineering, University of Utah USA

## Abstract

Silica nanoparticles (SNPs) are attractive for the delivery of drugs and as imaging agents due to their ease of synthesis and scale up, robust structure, and controllable size and composition. Degradability is one important factor that limits biomedical applications of SNPs. With this in mind, we designed, prepared and characterized novel hydrolysable organosilica nanoparticles (ICPTES–sorbitol SNPs). These particles were prepared by co-condensation of tetraethoxysilane with a bridged sorbitol-based silsesquioxane precursor containing carbamate linkages. The non-porous spherical ICPTES–sorbitol SNPs became porous after they were placed in an aqueous environment as a result of the hydrolysis of carbamate bonds and were completely degraded upon prolonged exposure to water. The rate of degradation depended on the pH of the solution, with nanoparticles degrading slower at pH 2 than at pH 4 or pH 7. The degradation was demonstrated by transmission electron microscopy, nitrogen desorption analysis and solution analytical techniques such as ICP-MS and molybdenum blue assay, which was also used to follow the dissolution of ICPTES–sorbitol SNPs.

## Abstract 2

We prepared novel water hydrolysable organosilica nanoparticles by co-condensation of tetraethoxysilane with a bridged sorbitol-based silsesquioxane precursor containing carbamate linkages.

## Graphical abstract

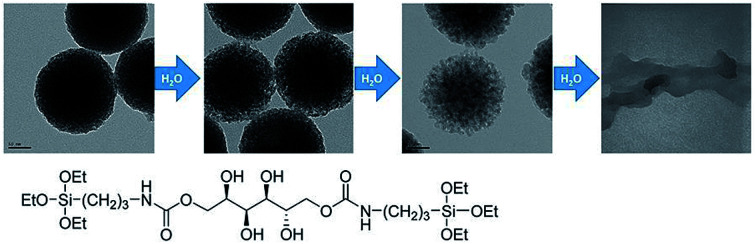

## Introduction

Silica nanoparticles (SNPs) are being explored in a wide variety of applications such as sensing,^1^ photonics,^2^ and catalysis.^3,4^ Of particular interest to our work are biomedical applications of SNPs as drug delivery^5–7^ and theranostics^8,9^ agents. Two common methods are used to prepare SNPs potentially suitable for such applications. Non-porous SNPs possessing various morphologies, chemical composition, and biological fate have been prepared by polycondensation of alkoxysilanes using the Stöber method.^10,11^ Surfactant-based techniques have been utilized to synthesize mesoporous SNPs with well-defined pore structure, controlled porosity, and high loading capacity.^12–16^

Since SNPs contain strong silicon–oxygen bonds, they cannot degrade easily inside the cells. This non-degradability of SNPs leads to acute and chronic toxicities^17–19^ due to the *in vivo* accumulation of SNPs in different organs such as liver, kidney, spleen, bladder, and lungs.^20–22^ Intact SNPs can induce oxidative stress (increasing the production of free radicals such as reactive oxygen species), lipid peroxidation, cell cycle arrest, apoptosis, genotoxicity, change mitochondrial membrane potential, and increase pro-inflammatory cytokines (*e.g.* TNF-α). It has been shown that the presence of surface moieties such as silanol groups (–SiOH) is also related to SNPs toxicity.^23^ This limits the potential utility of SNPs for *in vivo* administration. Hence, biodegradability of SNPs is a crucial factor in developing SNPs suitable for *in vivo* delivery. Several strategies have been developed to address the degradability problem of SNPs, as discussed below.

The second important consideration is the properties of SNP degradation products. This topic has been studied extensively in the past several years. It has been shown that the major product of SNP hydrolysis is monosilicic acid, Si(OH)_4_.^24–26^ Monosilicic acid is the soluble form of silica with the p*K*_a_ of *ca.* 9.8 (weak acid) containing silicon tetrahedrally coordinated to four hydroxyl groups.^27^ This molecule is nontoxic and can be transferred through the tissues, enter blood vessels, and eventually be excreted and cleared from the body through the urinary system due to its small size (<5.5 nm).^28^ Moreover, it has been shown that silicic acid derivatives are biocompatible and they have been found as trace elements in humans.^29^ In addition, studies have shown that silicic acid released from SNPs favours wound healing in human dermal fibroblasts (CCD-25SK cells).^30^ Our own recently reported experiments for biodegradable polysulfide-containing SNPs demonstrated that more than 85% cell viability can be achieved when RAW 264.7 macrophages were incubated with nanoparticle degradation products for 24 h.^31^ Therefore, it is safe to assume that as long as degradable SNPs comprise of silica and closely related materials their degradation products will be non-toxic, and the focus of the research effort should be on silica nanoparticles with improved degradability.

Earlier strategies to address the degradability problem of SNPs include the preparation of highly porous silica xerogels for controlled drug release^32,33^ and modification of the preparation conditions to produce biocompatible and bioresorbable sol–gel-derived SiO_2_ matrices.^34,35^ Another technique used to address this issue was the preparation of calcium-^36^ and iron(iii)-doped SNPs.^37^ Finally, biodegradable silica nanospheres have been prepared by incorporation of biodegradable polymers, such as poly(l-lactic acid)^38,39^ during nanoparticle formation.

Recently, a new approach to the preparation of degradable SNPs has been introduced. It is based on the incorporation of cleavable bonds into the silica matrix using silane precursors containing such bonds. For example, it has been reported that introducing disulfide (S–S) bridges within silica nanoparticles by co-condensation of tetraethyl orthosilicate (TEOS) and bis(triethoxysilylpropyl) disulfide leads to intracellular degradation of these nanoparticles when they are exposed to reducing agents such as glutathione (GSH).^31,40–42^ In our previous work^31^ we have shown that development of disulfide- and tetrasulfide-containing SNPs with a controlled degradation profile promises effective drug delivery with a predetermined carrier elimination profile. In that research, we synthesized a series of redox-responsive polysulfide-based biodegradable SNPs with low polydispersity and with variations in size (average diameters of 58, 108, 124, and 332 nm), porosity, and composition (disulfide *vs.* tetrasulfide bonds). The degradation kinetics of the nanoparticles was analysed in the presence of glutathione (GSH), mimicking the intracellular reducing conditions. Our results indicated that porosity and core composition play predominant roles in the degradation rate of these nanoparticles, with 108 nm mesoporous disulfide-based SNPs showing the highest degradation rate among all prepared nanoparticles. Another type of degradable SNPs incorporating cleavable bonds was also reported, prepared *via* sol–gel condensation reaction of an oxamide-bridged alkoxysilane (OBA) precursor with amide bonds, which could be cleaved *via* trypsin enzyme.^43^

However, to-date the preparation of SNPs which could be degraded in aqueous environment in the absence of additional reagents is still a challenge. In the present work, we describe the preparation and properties of water-degradable SNPs containing carbamate linkages. Specifically, we treated sorbitol with 3-isocyanatopropyltriethoxysilane (ICPTES) to obtain a bridged silsesquioxane containing two carbamate functionalities and used this silsesquioxane to prepare SNPs that degrade in water. Sorbitol used in this study as a part of the precursor is nontoxic and can be metabolized by the human body;^44^ hence hydrolysis of the ICPTES–sorbitol nanoparticles is not expected to induce adverse biological reactions.

## Experimental section

### Materials and instruments

Tetraethoxysilane (TEOS, 99.999+%, Alfa Aesar), 3-iso-cyanatopropyltriethoxysilane (ICPTES, Gelest), d-sorbitol (Sigma-Aldrich), sodium silicate solution (27.0% SiO_2_, Riedel-de Haën), and ammonium molybdate tetrahydrate (Alfa Aesar) were used as received. Triethylamine (Sigma-Aldrich) was freshly distilled. *N*,*N*-Dimethylformamide (DMF, Mallinckrodt) was dried by anhydrous magnesium sulphate (Mallinckrodt). Methanol (ACS Reagent, Sigma-Aldrich) and ethanol (200 proof, ACS-grade, Pharmaco-Aaper) were used without further purification. 18 MΩ cm water used in all the experiments was obtained from a Barnstead “E-pure” water purification system.

The morphological characterization was carried out using scanning electron microscopy (SEM, FEI Nova NanoSEM) and transmission electron microscopy (TEM, FEI Philips Tecnai T-12). All electron microscopy samples were prepared by depositing the nanoparticles from aqueous or ethanolic suspensions onto silica nitride wafers for SEM or on holey carbon-coated Cu grids for TEM. The surface area and pore volume of SNPs were measured by nitrogen sorption, which was performed using a Micrometrics ASAP 2010 instrument at 77.3 K. All samples (∼0.1 g) were degassed in the degassing port of the adsorption apparatus at 80 °C for approximately 6 hours prior to the nitrogen sorption measurements. The surface area was determined based on Brunauer–Emmett–Teller (BET) calculation method with relative pressure range between 0.05 and 0.25 and the pore size distribution was obtained using Barrett–Joyner–Halenda (BJH) method. UV-Vis measurements were performed using an Ocean Optics USB4000 instrument in a quartz cuvette. The nanoparticle hydrodynamic radii and zeta potential measurements were conducted in water using a NICOMP 380 ZLS Zeta Potential/Particle Sizer (PSS · NICOMP Particle Sizing Systems) at room temperature. Thermogravimetric analyses were performed using a TA Instruments TGA 2950 Thermogravimetric Analyzer under an N_2_ atmosphere from 35 to 800 °C at a heating rate of 20 °C min^−1^. Fourier transform infrared (FT-IR) spectrometer (Thermo Scientific Nicolet 8700 model) was used for the detection of functional groups. A Clay Adams Compact II Centrifuge (3200 rpm, Becton Dickinson) and ultracentrifuge Sorvall RC5B Plus (15 000 rpm on a SA-600 rotor) were used for all centrifugations.

### Synthesis of ICPTES–sorbitol silsesquioxane precursor

**Fig. 1:**
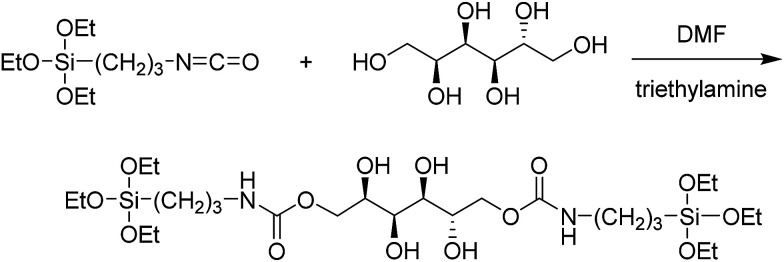
Synthesis of ICPTES–sorbitol silsesquioxane.

The sorbitol-bridged precursor was prepared (Fig. 1) following a published procedure.^45^ Briefly, 0.2 g of sorbitol was added to 3 mL of dry DMF in a round bottom flask using continuous flow of nitrogen and placed in an oil bath under constant stirring. The temperature was elevated to 40 °C to accelerate the dissolution of sorbitol. When sorbitol fully dissolved in DMF, 306 μL of freshly distilled triethylamine and 542 μL of ICPTES were added to the solution. The temperature of the solution was maintained at 90 °C for 3 days. The product (designated as ICPTES–sorbitol) was collected by solvent removal and washing with ethanol and acetone as pale yellow solid. Fig. S1† shows ^13^C and ^1^H NMR spectra of ICPTES and ICPTES–sorbitol.

### Synthesis of ICPTES–sorbitol SNPs

A typical ICPTES–sorbitol SNP synthetic procedure was as follows: 0.04 g of ICPTES–sorbitol was dissolved in 10 mL of ethanol followed by sonication for 1 h. Any remaining undissolved solids were excluded from the reaction by using the clear supernatant. 5 mL of NH_4_OH were added to this supernatant followed by the addition of 0.06 mL of TEOS under vigorous stirring. The onset of turbidity after a short while indicated the start of SNP formation. The reaction was conducted at room temperature for 2 h. The obtained nanoparticles were collected by centrifugation and washed with ethanol and water. The control SNPs were synthesized under the same reaction conditions without the addition of ICPTES–sorbitol. All resulting nanoparticles were air-dried and stored as a powder. Table S1† provides the different preparation conditions that were examined to optimize the ratio of the precursors, solvent, and catalyst.

### Degradation of carbamate-bridged SNPs

The ICPTES–sorbitol SNPs were suspended in aqueous solutions of pH 4 and pH 2 (HCl) and aliquots of nanoparticles were then periodically collected by centrifugation for 20 min at 15 000 rpm, and washed 4 times with ethanol and deionized (DI) water to remove HCl. The collected nanoparticles were then characterized by scanning electron microscopy (SEM), thermogravimetric analysis (TGA), transmission electron microscopy (TEM) and nitrogen adsorption–desorption. The degradation product of the nanoparticles, Si(OH)_4_, was detected in the supernatant by the reaction with molybdic acid leading to yellow silicomolybdate. To increase the spectrophotometric sensitivity, the silicomolybdate was further reduced by hydrated ammonium iron sulfate to yield Silicomolybdenum Blue complex. The supernatants were then analyzed using ultraviolet-visible (UV-Vis) spectrometer at 810 nm. Aliquots of the supernatant were also analyzed using ICP-MS (Agilent 7500ce) with cesium internal standard.

## Results and discussion

### Preparation of ICPTES–sorbitol SNPs

**Fig. 2:**
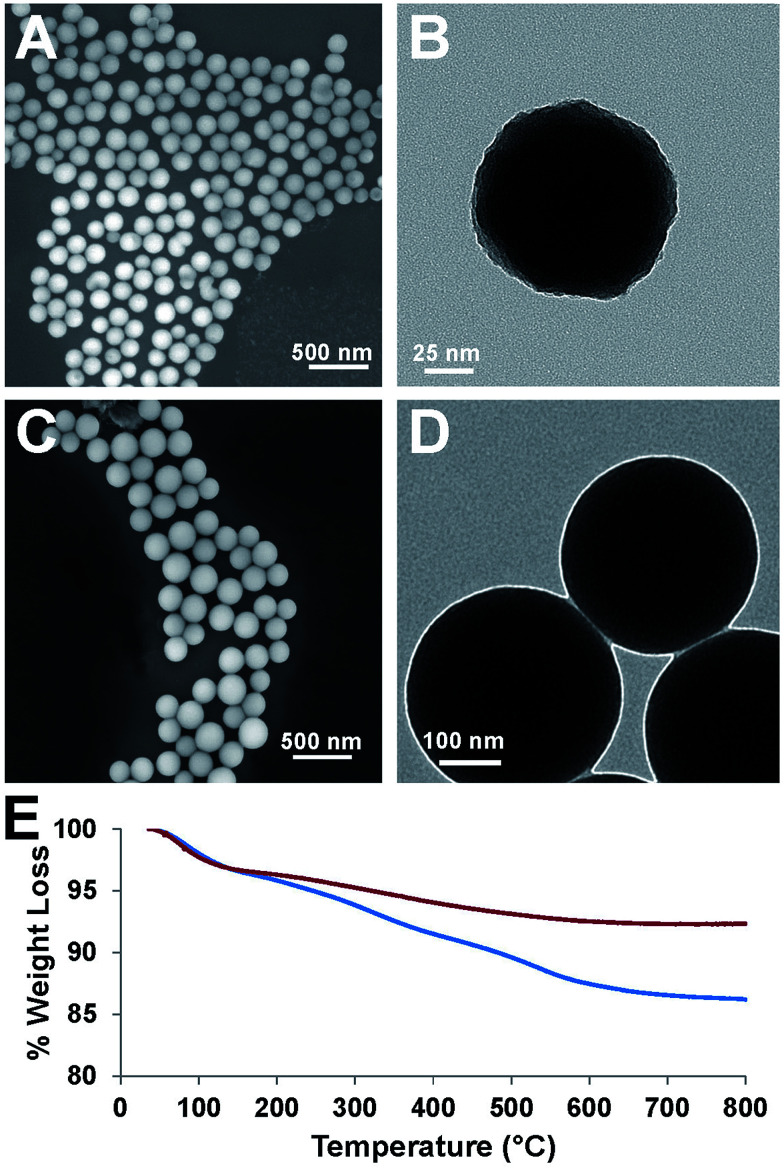
SEM and TEM images of (A and B) ICPTES–sorbitol SNPs and (C and D) SNPs produced under the same conditions using TEOS as the only silica precursor. (E) TGA plot for ICPTES–sorbitol SNPs (blue) and TEOS SNPs (red).

Our attempt to use ICPTES–sorbitol as a sole nanoparticle precursor did not lead to the formation of the corresponding nanoparticles. Thus, the preparation of ICPTES–sorbitol SNPs was achieved by hydrolysis and co-condensation of ICPTES–sorbitol precursor with tetraethyl orthosilicate (TEOS) under basic catalysis conditions. We used the modified Stöber method to prepare monodisperse silica spheres (Fig. 2A and B).^46^ The optimal reaction time was 2 hours with longer reaction times leading to aggregation of the nanoparticles. This suggests that the nanoparticles are formed in the first 2 hours, after which excess silica precursors condense loosely on the surface of the nanoparticles which leads to forming interconnections between the nanoparticles.

We found that the ratio between TEOS and ICPTES–sorbitol plays a critical role in forming spherical nanoparticles, with the shape is mainly dependent on TEOS concentration. Using high amounts of ICPTES–sorbitol precursor prevented the formation of uniform spherical nanoparticles (Fig. S2†). On the other hand, too much TEOS led to the formation of nanoparticles with limited degradability. After multiple trials, we found that the best ratio between the ICPTES–sorbitol and TEOS is approximately 1 : 5, leading to the formation of uniform nanospheres with the largest proportion of the organosilane as the degradable component. The formation of 122 ± 12 nm spherical non-porous nanoparticles was confirmed by SEM (Fig. 2A) and TEM (Fig. 2B). As a control, spherical SNPs were synthesized under the same reaction conditions as ICPTES–sorbitol SNPs by utilizing TEOS as the only silica source. These nanoparticles had the average diameter of 340 ± 29 nm (Fig. 2C and D) which was significantly larger than that of the hybrid ICPTES–sorbitol–TEOS SNPs.

TGA measurements confirmed the incorporation of organic groups in the ICPTES–sorbitol SNPs (Fig. 2E). In these experiments, the weight loss above 150 °C can be attributed to the calcination of the organic groups. The weight loss difference of 6.2% between ICPTES–sorbitol SNPs and TEOS SNPs suggested that ICPTES–sorbitol was incorporated into the silica network of the ICPTES–sorbitol SNPs. Assuming that all of 6.2% weight loss difference results from the organic substituents, 1.5 mol% of ICPETS–sorbitol was incorporated into the hybrid SNPs. The incorporated amount of organic groups was much lower than that calculated based on the initial molar ratio of ICPTES–sorbitol/TEOS used in the reaction. We speculate that the excess of ICPTES–sorbitol remained in solution and was removed after centrifugation and washing of the particles.

### Degradation of ICPTES–sorbitol SNPs in water at neutral pH

**Fig. 3:**
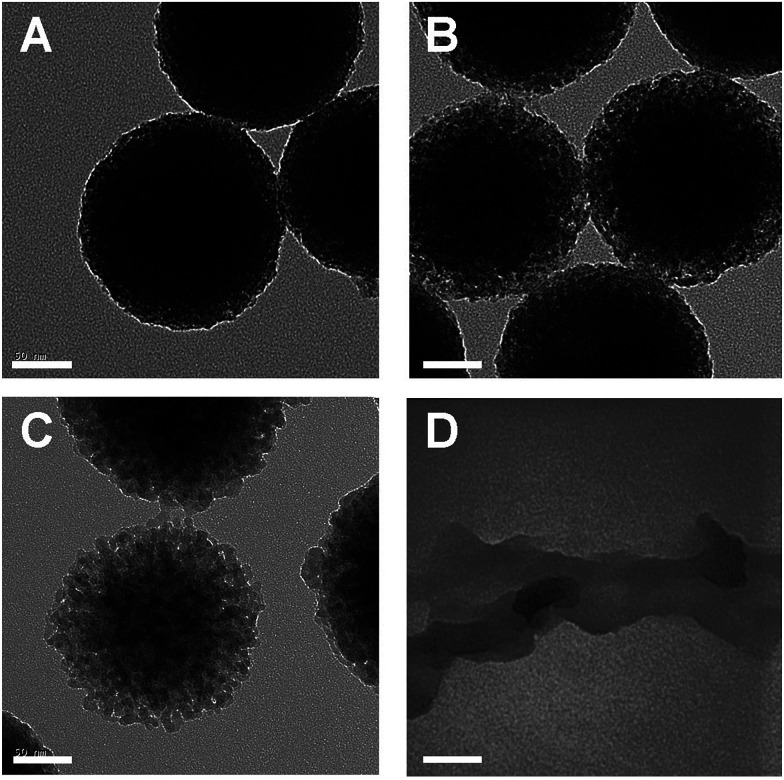
TEM images for ICPTES–sorbitol SNPs immersed in water (A) day 1, (B) day 5, (C) day 21, (D) day 90. Scale bars are 50 nm.

Degradation of ICPTES–sorbitol SNPs was first studied in water at neutral pH and TEM was used to observe the structural changes in the ICPTES–sorbitol (Fig. 3). While the as-made ICPTES–sorbitol SNPs were non-porous with slightly rough surfaces (Fig. 2B), they became porous after prolonged immersion in water. Instead of being hydrolyzed from the external surfaces, the spherical ICPTES–sorbitol SNPs initially maintained their general shape and size and hydrolysis occurred inside the spheres creating porosity (Fig. 3A–C). In contrast, the TEOS SNPs retained their morphology and non-porosity even after being kept in water for a month. After a month in water, ICPTES–sorbitol SNPs started to lose mass integrity and completely degraded after three months (Fig. 3D). This slow degradation may be related to the saturation of silica species in solution. A faster degradation behavior might be observed in a system with a continuous flow of water.

**Fig. 4:**
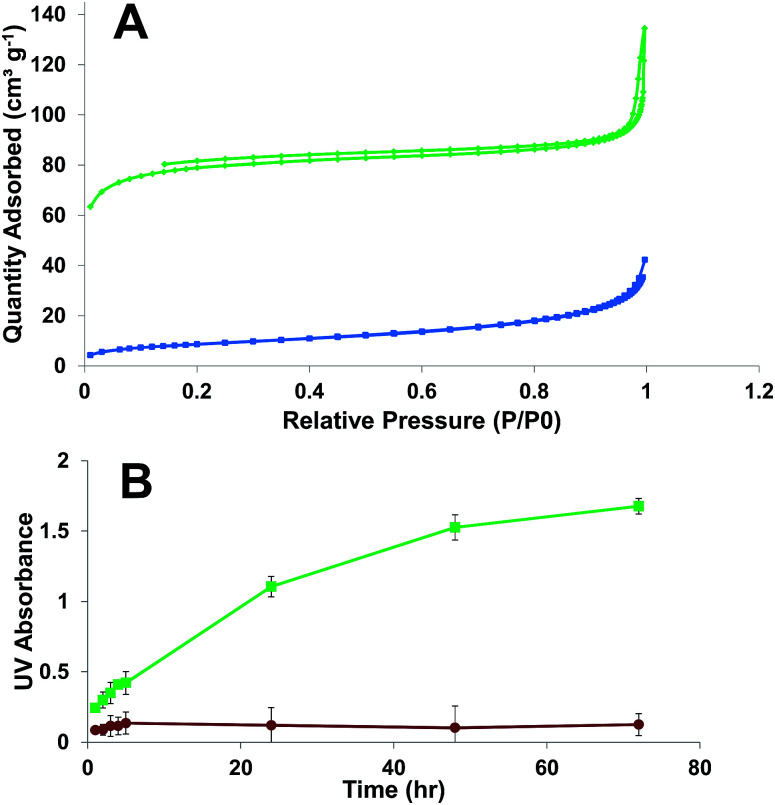
(A) Nitrogen adsorption desorption isotherms of as-made ICPTES–sorbitol SNPs (blue) and hydrolysed ICPTES–sorbitol SNPs (green) after immersion in water for 21 days, (B) Molybdenum Blue assay results for the dissolution of ICPTES–sorbitol SNPs (green) and TEOS SNPs (red) in DI water.

Nitrogen adsorption–desorption analyses for ICPTES–sorbitol SNPs before and after hydrolysis were carried out to measure the Brunauer–Emmett–Teller (BET) specific surface area and the Barrett–Joyner–Halenda (BJH) pore size distribution (Fig. 4A). The adsorption isotherm for as-made ICPTES–sorbitol SNPs exhibited Type III isotherm corresponding to non-porous nanoparticles. In contrast, a Type IV isotherm was observed for ICPTES–sorbitol SNPs after hydrolysis in water indicating a mesoporous structure. There were two hysteresis loops in the isotherm of hydrolyzed ICPTES–sorbitol SNPs. The hysteresis loop at lower relative pressure from ∼0.2 to 0.8 *P*/*P*_0_ was attributed to the filling of the mesopores and showed wide distribution of the pore size. The second hysteresis loop located at ∼0.9 *P*/*P*_0_ was due to the inter-particle aggregation. BET specific surface area was 31.0 and 266.5 m^2^ g^−1^ for ICPTES–sorbitol SNPs before and after hydrolysis, respectively. The hydrolyzed nanoparticles displayed a broad distribution of pore sizes with approximate diameter of 7.1 nm.

In order to follow the degradation process and to detect the hydrolysis products of ICPTES–sorbitol SNPs we used two analytical techniques commonly used for quantitative analysis of silicon. First, we examined the aqueous solutions where known amounts of ICPTES–sorbitol SNPs were placed at several time points using inductively coupled plasma mass spectrometry (ICP-MS). After the removal of the undissolved nanoparticles by centrifugation the aqueous solutions contained silicon with amounts increasing proportionally to the hydrolysis time. After complete degradation of ICPTES–sorbitol SNPs, these solutions contained silicon in quantities corresponding to the initial amounts of ICPTES–sorbitol SNPs.

Secondly, we used a well-established Molybdenum Blue colorimetric method to demonstrate the formation of monosilicic acid in the course of the degradation of ICPTES–sorbitol SNPs and to quantify its amount.^42–47^ The supernatants obtained as described above were treated with molybdic acid resulting in the formation of a yellow solution, which became blue upon the addition of ammonium iron sulfate. These reactions are characteristic of monosilicic acid conversion to silicomolybdate which is reduced by ammonium iron sulfate to yield Silicomolybdenum Blue complex. In addition to using this method to confirm the formation of monosilicic acid we used it to follow the hydrolysis of ICPTES–sorbitol SNPs as a function of time. Fig. 4B shows that the dissolution/hydrolysis of ICPTES–sorbitol SNPs was much faster in comparison to the Stöber nanoparticles, which did not dissolve in water. The amounts of Silicomolybdenum Blue complex after the complete degradation of ICPTES–sorbitol SNPs were determined by the spectrophotometric method and were found to be in good agreement with the initial amounts of ICPTES–sorbitol SNPs.

### Degradation of ICPTES–sorbitol SNPs under acidic conditions

**Fig. 5:**
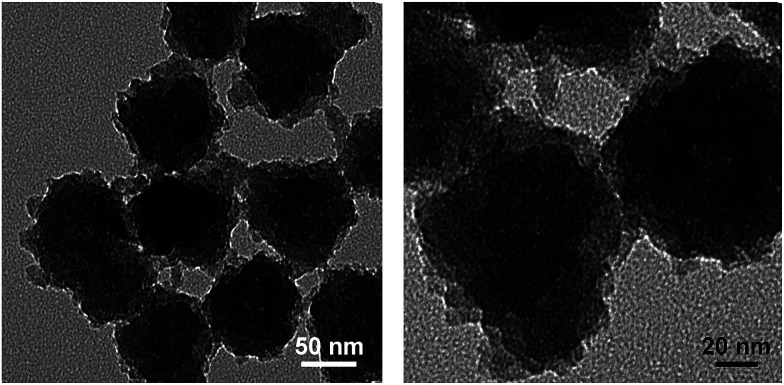
TEM images for ICPTES–sorbitol SNPs hydrolysed at pH 4 for 21 days (scale bars 50 and 20 nm).

Next, we studied the degradation of ICPTES–sorbitol SNPs at pH 2 and 4. TEM images of the nanoparticles hydrolyzed in HCl aqueous solution at pH 4 showed that ICPTES–sorbitol SNPs broke into irregularly shaped pieces under the acidic conditions after 3 weeks (Fig. 5) and completely dissolved after 3 months (TEM results were analogous to that shown in Fig. 3D). However, when hydrolyzed in HCl aqueous solution at pH 2, the nanoparticles became porous but the shape of the particles were not as irregular as those hydrolyzed at pH 4 (Fig. S3†). We found that when ICPTES–sorbitol SNPs were immersed in simulated body fluid (SBF) at pH 7.4 they underwent degradation in the same manner as when they were immersed in acidic water (Fig. S4†). We hypothesize that the degradation of ICPTES–sorbitol SNPs in SBF may be accelerated by the presence of ions, as previously reported.^48^

We compared the zeta potential of ICPTES–sorbitol SNPs before and after their exposure to aqueous HCl solution at pH 4. The negative zeta potential value for ICPTES–sorbitol SNPs (−44.3 ± 0.5 mV) became positive (+42.0 ± 0.8 mV) after 12 days, which suggested the formation of positively charged groups on the SNPs surface. This might result from carbamate hydrolysis inside ICPTES–sorbitol SNPs leading to sorbitol, CO_2_ and 3-aminopropyl chains attached to the silica matrix (Fig. S5†). In order to verify this hypothesis, a colorimetric experiment was conducted. Dansyl chloride forms a green fluorescent adduct with primary amines. We treated ICPTES–sorbitol SNPs and Stöber SNPs, which were immersed in solution of different pH values for a week, with dansyl chloride. After the addition of dansyl chloride solution, the ICPTES–sorbitol SNPs hydrolyzed at pH 4–8 exhibited green fluorescence confirming the presence of primary amines on the surface of the nanoparticles. On the other hand, both ICPTES–sorbitol SNPs hydrolyzed at pH 2 and Stöber particles showed no fluorescence (Fig. S6†).

To better understand the extent of acidic hydrolysis, the as-made ICPTES–sorbitol SNPs and those hydrolyzed at pH 2 and 4 for a week were examined gravimetrically. The weight loss of the hydrolyzed ICPTES–sorbitol SNPs was *ca.* 30% at pH 2 and 53% at pH 4. This confirmed that pH greatly affects the degradation of these nanoparticles and more extensive degradation occurs at pH 4 compared to pH 2.

The slower hydrolysis of ICPTES–sorbitol SNPs at pH 2 demonstrated by TEM imaging, dansyl chloride test, and gravimetric analysis is in agreement with the earlier report^49^ that silica particles are resistant to degradation at very acidic conditions. One may take advantage of this difference in ICPTES–sorbitol SNPs degradation rates in oral-based drug delivery applications, where these nanoparticles may be able to withstand the highly acidic environment of the stomach but may be hydrolyzed in the neutral to slightly acidic environment of the intestines.

## Conclusions

In summary, we prepared unique hydrolysable silica nanoparticles (ICPTES–sorbitol SNPs) by the incorporation of carbamate linkages into the silica matrix. For this purpose, a silsesquioxane ICPTES–sorbitol containing carbamate groups was synthesized and used to produce the novel nanoparticles by co-condensation with tetraethoxysilane. Several characterization methods were used to confirm that ICPTES–sorbitol SNPs completely degraded in water at neutral and acidic pH as the result of the carbamate linkage hydrolysis with monosilicic acid as the main degradation product. Our recent studies of degradable silica nanoparticles as well as those of others demonstrated that monosilicic acid is nontoxic to cells and organs, and can be transferred through the tissues, enter blood vessels, and eventually be excreted through the urinary system. Therefore, it can be confidently predicted that the novel ICPTES–sorbitol SNPs are suitable to become superior building block for future theranostic agents.

In addition, the obtained novel SNPs became porous and formed primary amines on the surface in the process of their degradation, which could be of interest for future applications of these constructs as multifunctional nanoparticles for drug delivery and imaging. For example, anticancer drugs unstable in aqueous environment may be incorporated inside these nanoparticles during their preparation and would be protected from the aqueous environment for a period of time. When pores are formed by hydrolysis of the carbamate linkages inside the nanoparticles the drug may be released in the target organ. In addition, the novel SNPs showed different hydrolysis rates at pH 2 and 4, which may be of interest in oral-based drug delivery. While drug loading and release studies was beyond the scope of the present work, research in these directions is currently under way in our laboratories.

## Conflicts of interest

There are no conflicts to declare.

## Supplementary Material

RA-008-C7RA12377A-s001
