# Supplementary material for: Synthesis of water-degradable silica nanoparticles from carbamate-containing bridged silsesquioxane precursor
Source: RSC Adv. 2018 Jan 29;8(9):4914–20. doi: 10.1039/c7ra12377a (PMC6133298; doi:10.1039/c7ra12377a)
Supplement: RA-008-C7RA12377A-s001 [file RA-008-C7RA12377A-s001.pdf]

Electronic Supplementary Material (ESI) for RSC Advances.
This journal is © The Royal Society of Chemistry 2018

Electronic Supplementary Information

Synthesis of water-degradable silica nanoparticles from carbamate-containing bridged silsesquioxane precursor

Zhe Gao,[a] Seyyed Pouya Hadipour Moghaddam,[b, c] Hamidreza Ghandehari,[b,c,d] and Ilya Zharov[a,*]

Contents

1. Molybdenum blue colorimetric experiment
2. Supplementary table
3. Supplementary figures

1. Molybdenum Blue colorimetric experiment

All the solutions were contained in polyethylene bottles. Standard silicic acid solution was made by diluting sodium silicate solution ($Na_2Si_3O_7$, 242.23 g/mol). Three solution stocks were prepared: (1) 3.1 g of ammonium molybdate tetrahydrate dissolved in 50 mL of 1 M sulfuric acid, (2) 6.3 g of oxalic acid dihydrate dissolved in 50 mL of Millipore water, and (3) 1.76 g of ascorbic acid dissolved in 50 mL of Millipore water. Sonication was used to help dissolve the salts. In order to make a calibration curve, the standard silicic solutions with concentrations of 1.72×10^{-5} M, 8.56×10^{-5} M, 1.703×10^{-4} M, 2.54×10^{-4} M, 3.37×10^{-4} M, and 4.195×10^{-4} M were prepared, respectively. 100 μL of solution 1 was added to 2 mL of the sample. After 10 min, the solution became yellow due to the formation of yellow phosphate molybdenum complex. 100 μL of solution 2 was then added and the yellow color faded in 1 min. The addition of 100 μL of solution 3 resulted in a color change to blue. The mixture was allowed to sit for an additional 10 min until the completion of the blue complex formation. The UV-vis spectrum featured a molybdenum blue peak at 810 nm. The concentration of silicic acid was calculated from the calibration curve. 10 mg of 340 nm Stöber SiNPs and ICPTES-sorbitol SiNPs were each separately dispersed in 20 mL of Millipore water. An aliquot of each solution was centrifuged every hour. 100 μL of each supernatant was diluted by 2 mL of water, and the resulting solutions were tested by the procedure aforementioned.

2. Supplementary table

Table S1. The reaction conditions for fabrication of ICPTES-Sorbitol SiNPs

Run No.	ICPTES-Sorbitol (mol) $\times 10^{-4}$	TEOS (mol) $\times 10^{-3}$	Ethanol (mL)	NH_4OH (mL)	Time (hr)
1	3.0	0.68	10	5	18
2	3.0	1.4	10	5	18
3	3.0	4.5	10	5	18
4	3.0	4.5	11.25	3.75	18
5	3.0	9.0	12	4	18
6	0.30	0.14	10	5	2
7	0.60	0.28	10	5	2

3. Supplementary figures

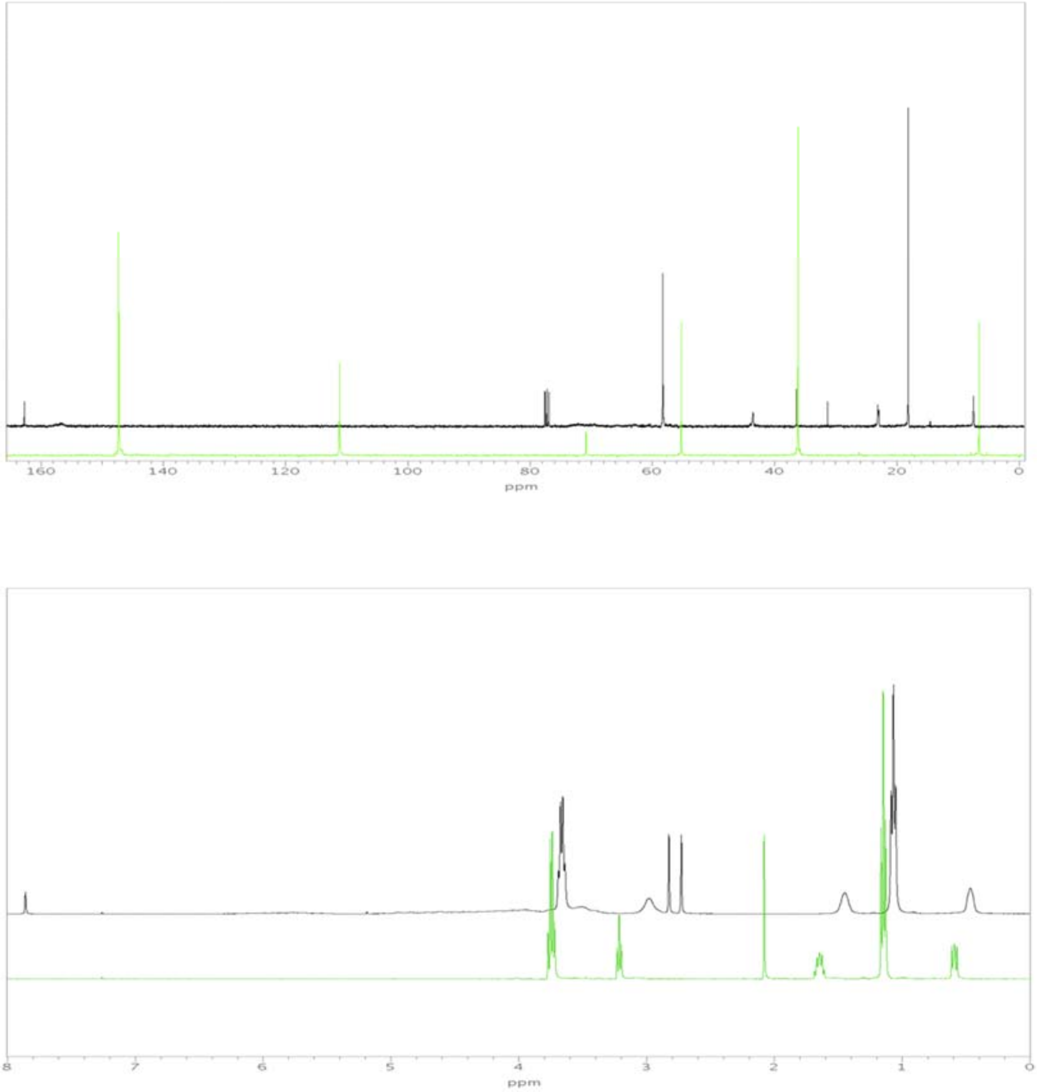

Fig. S1. ^{13}C NMR (Top) and H NMR (Bottom) overlay spectra of ICPTES (Green) and ICPTES-Sorbitol (Black).

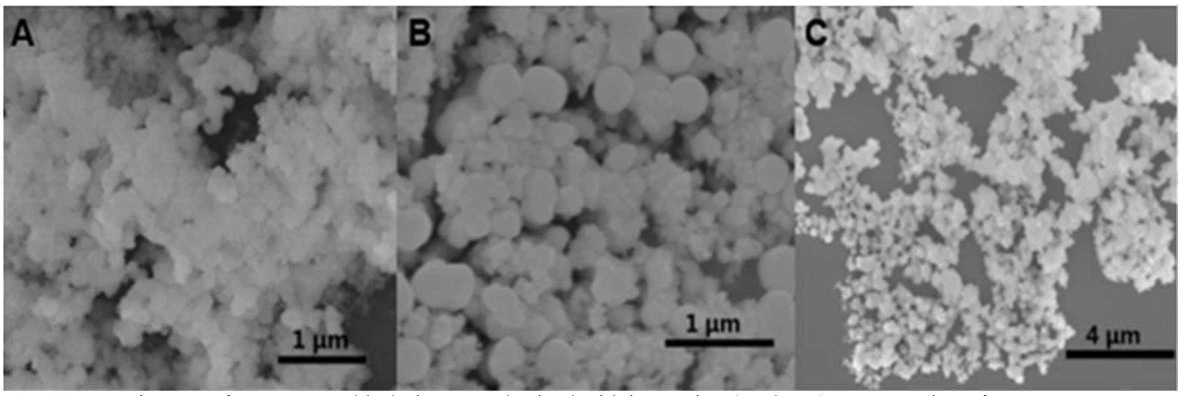

Fig. S2. SEM images of ICPTES-Sorbitol SiNPs synthesized with increasing (a < b < c) concentration of TEOS.

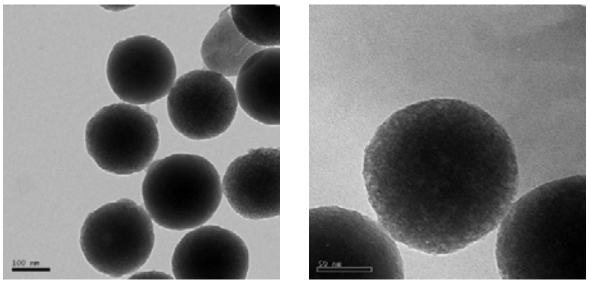

Fig. S3. TEM images of the nanoparticles hydrolysis in water (HCl, pH 2) after 3 weeks.

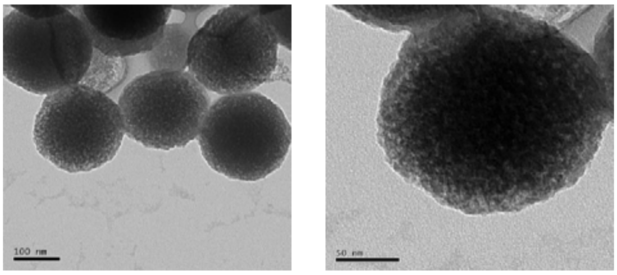

Fig. S4. TEM images of the nanoparticles hydrolysis in SBF (pH 7.4) after 3 weeks.

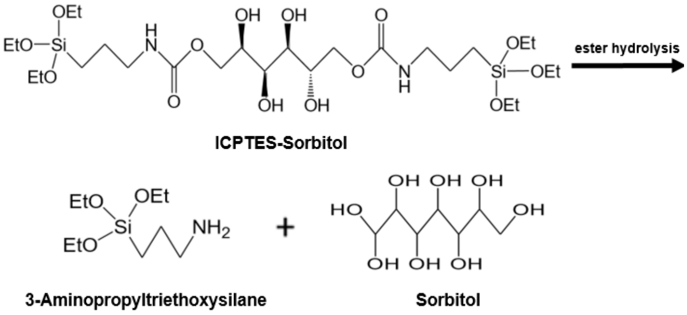

Fig. S5. Proposed hydrolysis mechanism of ICPTES-Sorbitol.

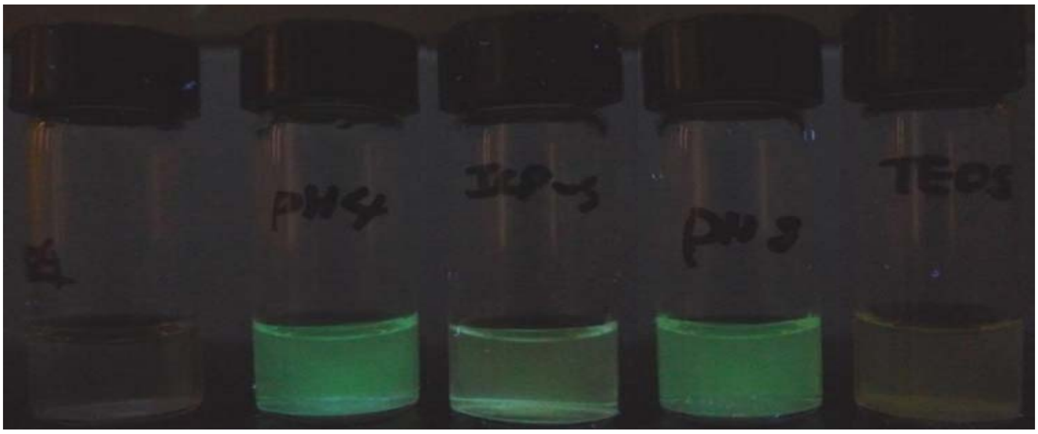

Fig. S6. Dansyl chloride reacted with primary amines from the hydrolysis of the nanoparticles (left to right): ICPTES-Sorbitol SiNPs at pH 2, pH 4, pH 7, pH 8, TEOS SiNPs at pH 7.
